# Critical discussion on a method for derivation of reference limits in clinical chemistry from a patient population

**DOI:** 10.1155/S1463924685000062

**Published:** 1985

**Authors:** J. B. Hemel, F. R. Hindriks, W. van der Slik

**Affiliations:** Central Laboratory for Clinical Chemistry University Hospital Groningen P.O. Box 30001 Groningen 9700 RB The Netherlands


Journal of Automatic Chemistry, Volume 7, Number 1 (January–March 1985), pages 20–30

### Critical discussion on a method for derivation of reference limits in clinical chemistry from a patient population

**J. B. Hemel, F. R. Hindriks and W. van der Slik**
*Central Laboratory for Clinical Chemistry, University Hospital Groningen, P.O. Box 30001, 9700 RB Groningen, The Netherlands*

### Introduction

'Health' is a concept that is difficult to define. A well-known definition is given by the World Health Organization (WHO): health is a state of complete physical, mental and social well-being, and not merely the absence of disease or infirmity. Some authors, for example Gräsbeck [1], have criticized this definition as unrealistic and have proposed others that stress the absence of undesirable affections, the so-called privative definitions. A more complete discussion of the notion 'health' is beyond the scope of this paper.

All writers agree that health is a very individual state, so it seems inappropriate to define it by 'normal values' in clinical chemistry. However, because it is not generally possible to specify normal value for individuals, some form of limit for detecting gross deviations from 'health' would be useful. Of course no limit should be taken as absolute and it should not be called a 'normal' value, but, rather, a 'reference' limit. When determining reference limits it is important to ensure that the reference population matches the patient population as closely as possible in all respects other than disease. Special attention must be paid to similar age and sex distributions, social backgrounds, diets etc. Of course the same sampling and analysis methods must be used for both populations. Since blood donors or laboratory staff usually at least do not have the same age distribution as hospitalized patients, large discrepancies may be found if the former groups are used as reference samples. The International Federation of Clinical Chemistry (IFCC) has given a series of recommendations on the theory of reference values [2]. Solberg has designed a computer program package (REFVAL) to assist in the statistical treatment of reference values.

It would be very helpful if hospital patients could be their own references, or rather their colleagues' ones. This might well be possible if we assume that most patients have only a few distrubed chemical parameters and are 'healthy' in terms of the others. Then, in the bulk of analyses, most results will be 'normal' and the distribution will be polluted by pathological results only to a small extent. This paper describes an attempt to filter out these pathological values.

It must be noted that the benefit of being able to establish normal ranges that are more or less universal is reduced by this approach, because most patient populations will not sufficiently match the one the reference limits are determined for. Furthermore, there will be a slight variation in reference intervals determined at different times in the same hospital. The authors consider the gain in usefulness of reference intervals to be greater than these disadvantages.

### Materials and methods

### *Apparatus*

Analyses were performed on two Technicon SMA-C continuous-flow analysers that determine sodium, potassium, chloride, urea, creatinine, uric acid, alkaline phosphatase (AP), lactate dehydrogenase (LDH), aspartate aminotransferase (ASAT), alanine aminotransferase (ALAT), total bilirubin, direct bilirubin, calcium, inorganic phosphate, total protein, albumen, cholesterol, triglycerides, iron and gamma glutamyl transferase simultaneously in serum. For every sample analysed by an SMA-C all components are measured, due to the concept of multichannel analysis. In this study this was essential because the majority of the measurements on each component came from patients in whom most components were not pathological.

One SMA-C was for hospital patients and the other for general practitioners' patients. For data acquisition a Hewlett-Packard HP 1000 computer system was used. The program used for the calculations was written by Hemel in Pascal and was run on the University's Control Data Cyber 170/760 computer. The plots were made on the University's Versatec V80 electrostatic plotter.

### *Techniques*

Several methods have been devised in the past using the idea of deriving reference intervals from patient populations. Naus [3] has reviewed the following methods:

*Hoffmann* [4 and 5]: a graphical method using probability paper.

*Neumann* [6]: an iterative modification of the Hoffmann method.

*Pryce* [7]: mean and standard deviation based on the mode and percentiles of the non-distorted side of the one-sidedly polluted distribution.

*Becktel* [8]: a modification of the Pryce method.

*Bhattacharya* [9]: mean and standard deviation are derived from the intercept and slope of a plot of

$\ln (f(x+h)/f(x))$ against $x$, with $f(x)$: frequency in class with midpoint $x$, and $h$: class width.

Naus did not cover a method devised by Martin [10 and 11], who proposed population dissection by fitting two (or three) Gram-Charlier functions that represent a reference distribution, and a (two) polluting pathological one(s), to the observed distribution.

The method of choice should meet the following requirements:

(1) As the exact distribution of a variable is usually unknown, the method should preferably be nonparametric: most methods do not meet this requirement, they suppose a Gaussian distribution, if necessary after transformation. Martin's method does not make any assumption about the shapes of the populations. However, it supposes that the polluting pathological cases belong to the same one (or two) population(s).

(2) All pathological results should be filtered out: all methods try to do so. Martin's method places them in one (or two) separate distribution(s).

After an extensive study of the methods he reviewed, Naus advised the use of an automated and newly objective Bhattacharya method for known Gaussian distributions, and developed a method postulating a Pearson gamma distribution for distributions that are known to be non-Gaussian. Although Martin's method has the advantage of not making any assumptions about the shape of the distributions, reasonably good estimates for eight (or even 12) parameters are required to reach convergence of the iterative procedure. We expected this to be difficult to achieve. A further disadvantage is the fact that the pathological cases are thought to form a limited number of populations (one or two). As the choice between a gamma distribution and a Gaussian one was supposed to be usually simple, Naus's techniques were tested with data from the Central Clinical Chemical Laboratory.

*Variables that are approximately normally distributed*

The technique used was Naus's modification of the Bhattacharaya method [3 and 9]. A plot of $\ln (f(x+h)/f(x))$ against $x$ (with $f(x)$: frequency in class with midpoint $x$, and $h$: class width), gives a virtually straight line for a normal distribution, presented as a histogram (see Appendix A). A sample from a normal distribution that is partially (only in the tails) distorted by pathological cases will show a straight part in the plot for the 'clean' fraction of the sample (figure 1). It is possible to estimate the population mean and variance from the $x$-intercept and the slope, respectively. The problem of isolating the straight part from the zig-zagging ones remains, this is generally done by intuition. Naus proposed weighting factors to reduce the influence of the distorted parts on the regression line. Initially, wanted influences of two kinds are accounted for in the weighting:

(1) Random fluctuations: these affect the low-frequency bars of the histogram more than the high-frequency bars.

(2) Deviations from the wanted regression line because of pathological scores.

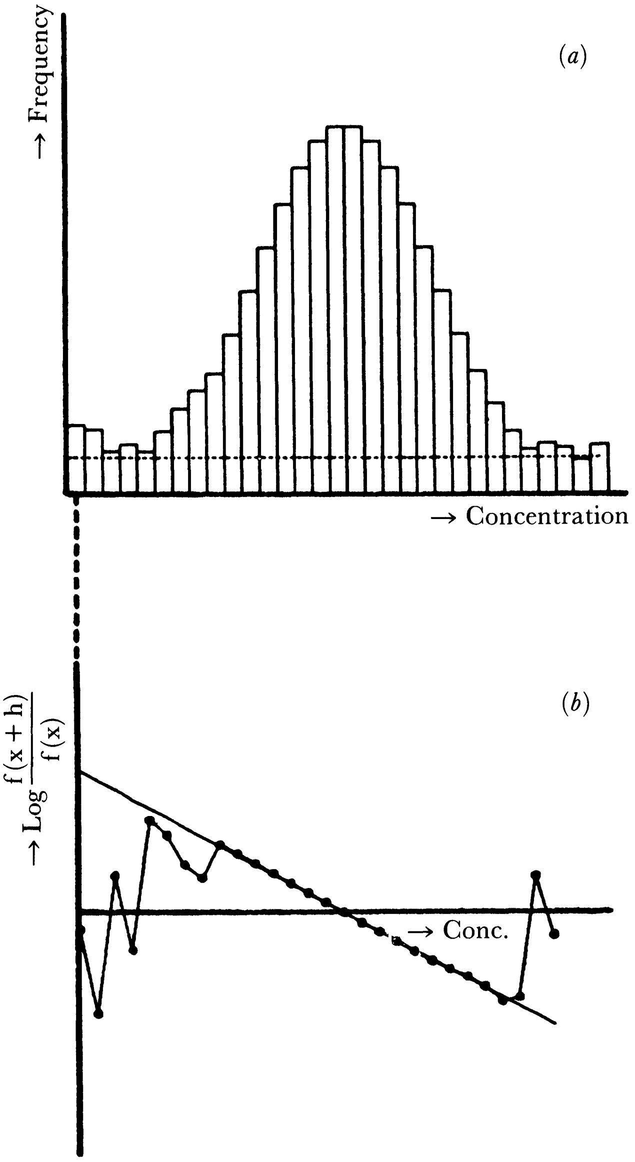

*Figure 1. An example of a Gaussian distribution that is polluted in the tails. Figure 1(a) shows the observed histogram and 1(b) shows the Bhattacharya plot belonging to it.*

Deviations of both kinds should have as little influence as possible on the regression line. Naus derived the following weighting factor formula:

$$w(x) = \frac{(f(x+h) + f(x))^{\alpha}}{(1 + d(x)/d(av))^{\beta}}$$

where $f(x)$ = frequency of class with midpoint $x$
$h$ = class width
$d(x)$ = deviation from regression line of class with midpoint $x$
$d(av)$ = average deviation from regression line
$\alpha$ and $\beta$ = unknown powers.

Empirically optimal results are found for $\alpha = 2$ and $\beta = 0$, so the weightings become:

$$w(x) = (f(x + h) + f(x))^2.$$

The statistical interpretation of these weightings is that the variance in the class frequencies is not equal for all classes and is balanced by the weightings.

By doing this, all short histogram bars (low frequencies) have minimal influence on the regression line. This method was tested on several artificially polluted Gaussian curves by trying to retrieve the parameters of the original curves. This gave excellent results. Subsequently, the author visually checked the results by inspecting the Bhattacharya plot and regression line of the smoothed histogram; the histogram is smoothed before making a Bhattacharya plot for visual inspection to get rid of the random fluctuations which mask the straight part in the plot. Smoothing can be done following the procedure described by Savitsky and Golay [13], and corrected by Steinier *et al.* [14].

However, there are some drawbacks in this approach:

(*a*) The top part of the histogram should be 'clean' (free from pathological results) because the weightings only include the frequencies. The situation shown in figure 2 is quite believable; ill patients form a distribution that is almost uniform. The resulting superposed (and observed) Bhattacharya plot will not show a straight part, although it might have a distortion that is not conspicuous, so that it could be overlooked (figures 3 [*a*] and [*b*]). In practice, more than one illness will disturb a 'healthy population'. It is difficult to predict how the straight line will be distorted. Even an exactly Gaussian distribution will not, in a practical sample, show a perfectly straight line because of random errors. So a seemingly straight line does not guarantee that there really is a Gaussian distribution, let alone that the observed slope and intercept give the right parameters. For these reasons the method is not adequate to test the hypothesis of dealing with a Gaussian distribution, for which it is often used. Statistically testing this hypothesis requires a homogeneous distribution, so it is difficult to do. The only way left is to base the usage of the method on prior knowledge about the shape of the distribution, for instance on calculations of coefficients of skewness and kurtosis. Of course, these calculations cannot be made for a mixed population such as the patient population used here.

(*b*) Paradoxically, disturbed parts of the plot have more influence on the regression line the more disturbed they are, because by pollution with pathological results their frequencies rise and consequently their weightings also increase.

(*c*) In principle, the range of the histogram influences the results because the tails 'pull with their weights' at the regression line. However, thanks to the small weightings on the tailing parts, this effect is small.

Therefore the part with higher frequencies is selected rather than the undisturbed part.

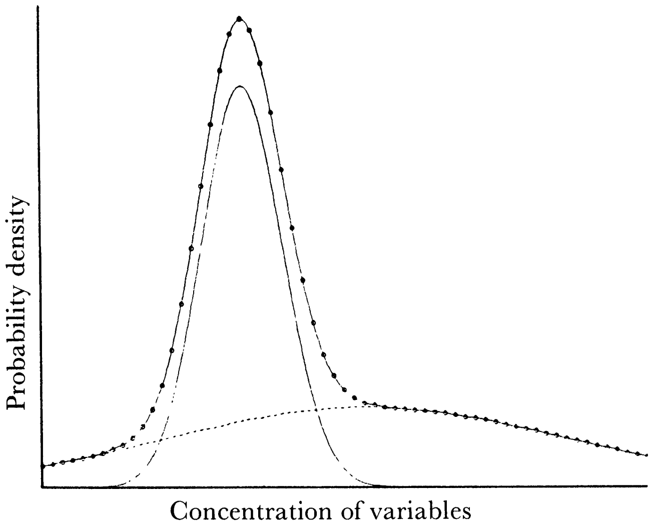

*Figure 2. A fictional situation in which a Gaussian distribution of 'healthy' people is distorted by adding another normal curve of patients, with a large variance. This results in an observed curve that is not readily recognized. The abscissa represents the concentration (for example in serum) of a relevant variable. Where ——— healthy population; - - - - - patients; - · - · - observed distribution.*

Some improvement could result from two modifications of the technique:

(i) A threshold of a fraction of the mode, for example one-tenth, to diminish the range effects.

(ii) An additional plot of residuals of unsmoothed results after linear regression analysis, in order to facilitate the detection of systematic deviations from the Gaussian model. An example is seen in figure 1. The Bhattacharya plot (figure 3[*b*]) appears to have a straight part, but the plot of residuals shows clearly that they are not randomly distributed about zero, so calculated reference intervals will be wrong (figure 3[*c*]).

*Variables that are definitely not normally distributed*

These variables are supposed to follow a Pearson's gamma distribution. This is a two-parameter distribution: $R$ for the number of degrees of freedom, and $\lambda$, which is an extension factor. A chi-square distribution with $\nu$ degrees of freedom is a gamma distribution with $R = 0.5*\nu$ and lambda $= 0.5$ (see Appendix C). Typical shapes of gamma distributions are illustrated in figure 4. These curves are skewed but tend to become symmetrical with many degrees of freedom.

The criteria for choosing the gamma curve as a model for a variable's distribution are:

(1) The distribution is known to be positively skewed (long tail to the right).

(2) The shape of the Bhattacharya plot is bent. (It should be straight if the distribution is normal.)

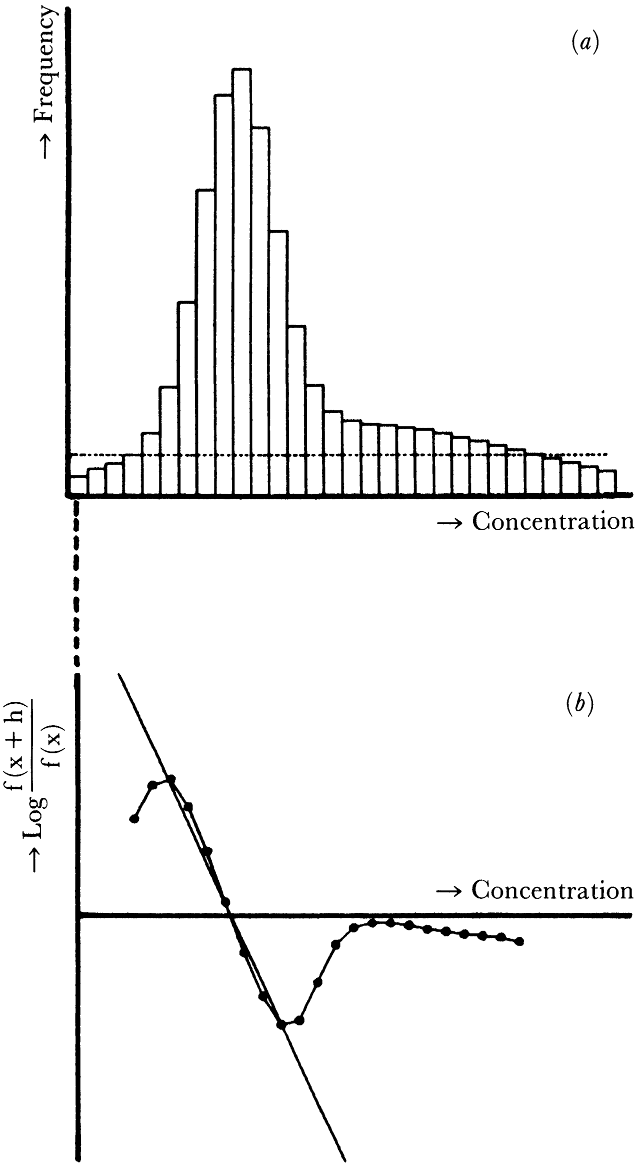

*Figure 3(a). A histogram of the situation described in figure 2. Where - - - threshold for calculations and plots.*

*(b) The Bhattacharya plot belonging to it, with the calculated regression line.*

*(c) The plot of residuals belonging to the Bhattacharya plot. This plot is drawn only for the reference interval estimated from the regression line to aid interpretation.*

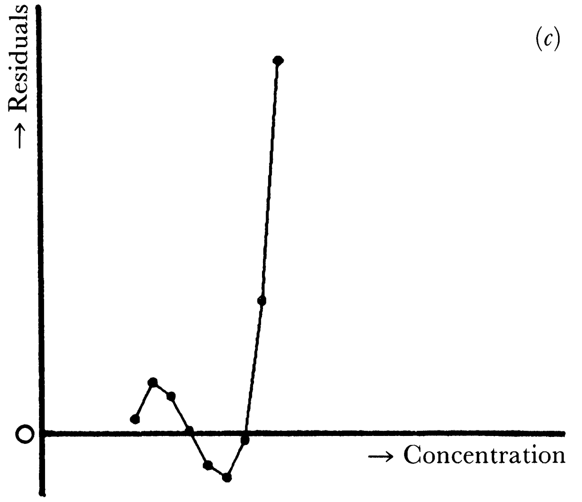

$R$ and $\lambda$ are numerically estimated using a weighted linear regression technique with the weightings as described above (the *procedure* of the numerical estimation of R and $\lambda$ is described in Appendix B). To establish upper (and lower) boundaries the desired fractiles are calculated from a chi-square distribution and transformed into a gamma distribution interval (see Appendix C).

*Comments*

In Naus's method the weightings for the linear regression technique are derived from experiments with a Gaussian distribution that is one-sidedly distorted by another Gaussian distribution. One might question whether this model is optimal for weighting gamma distributed variables.

In the method described, the 97.5% fractile is chosen as the upper boundary of a one-sided reference interval. The 95 percentile might be more appropriate.

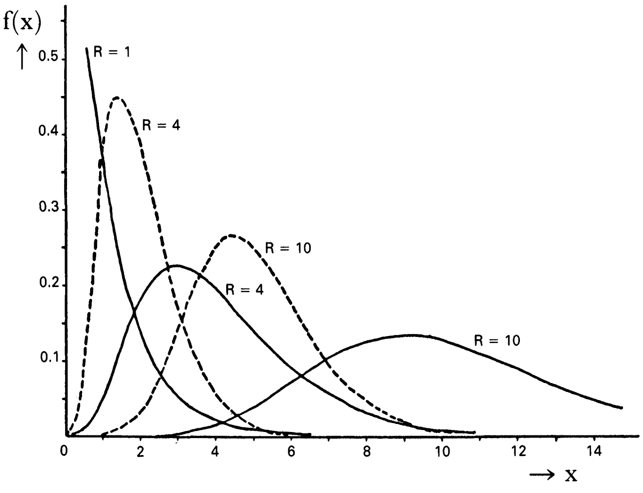

*Figure 4. Some typical shapes of gamma distribution curves. f(x) is the probability density. Where ——— $\lambda = 1$, - - - - - $\lambda = 2$.*

Again, the range of the histogram used to estimate the variable's distribution is of some importance for the results. So a threshold frequency of, say, one-tenth of the modal frequency could bring some improvement.

Testing the quality of the derived reference intervals is, of course, tedious. A way of checking is to compare them with literature values, preferably from the same laboratory; of course this is not a thorough test because these values are generally not based on a patient population. A better quality control would be some form of in-process checking: testing not the results, but, rather, the method. The model used can be judged visually by drawing a plot that should be a straight line in the case of a perfect gamma curve. The residuals can be plotted to determine whether there are trends visible in the deviation from the model. Details about these plots are given in Appendix B. In figures 5, 6 and 7 the function for deriving a straight line in the case of a gamma distribution is denoted by $F(\lambda, R, x)$.

### Discussion and results

Of course, the distributions of clinical chemical variables are not exactly Gaussian or gamma. However, one may hope that these distributions can be used as reasonably good approximations. In many cases, intuition and prior knowledge supports this idea. A major problem is finding a good criterion to choose between the models and to test the validity of the chosen model. Both methods should perhaps be used and the best chosen on the basis of interpretation of the plots of residuals. A major difficulty is the necessary weighted interpretation of the plots. Another criterion might be the estimated $R$ parameter (number of degrees of freedom) of the gamma approximation. If $R$ is greater than a certain number, for example 50, the distribution will be almost symmetrical and so a Gaussian model might be appropriate. Although this criterion may perform well in practice, it must be stressed that, theoretically, it is not a guarantee for the right choice. Fortunately, sometimes it is apparent that a distribution is symmetrical, so that the Gaussian model would be chosen, or positively skewed, that is, long tail to the right, in which a gamma model would be more appropriate.

Minor problems are the influence of the range of the histograms and the coarseness in the estimation of the interval that results. Reasons for these problems being minor are the arbitrariness of the commonly used central 95% interval and the individuality of 'health'.

Many clinical chemical variables are strongly influenced by such factors as age, sex, time and way of sampling. A partitioning of the reference patient group according to these parameters is necessary in order to avoid an 'averaged' reference interval, which would, of course, be worthless to a clinician. In some cases it may be necessary to incorporate the varying precision of analytical methods along the range of possible results in the weighting factors to reach optimal reference intervals. For some determinations, analytical precision is too small to resolve the model of the variable's distribution. (In our case this applied to the direct bilirubin determination.) Only variables used in screenings are fit for this method of deriving reference values, others are too strongly distorted by pathological results.

Results are given in tables 1 and 2. Choosing the best model must be done by the reader. A suggestion is given in the right-hand column (marked '5'). Some typical plots are given in figures 5, 6 and 7. Partitioning of the results according to sex, age etc. was impossible for us; and this means that some calculated reference values must be doubtful (table 2). It is often difficult to conclude from the calculations and plots whether the reference values found are valid or not; see, for instance, the cholesterol reference interval (table 2) and the plots relating to it (figure 7). The similarity between the reference intervals of (nearly) Gaussian distributed variables if they are based on a normal model and those based on a gamma model is remarkable. One might suggest that a gamma model provides for all cases, but in gamma distributions the variance ($=R/\lambda^2$) is dependent on the mean ($=R/\lambda$); this is unlikely to always be the case with clinical chemical variables, so the observed fact must be a coincidence. Proof that a reference interval applies only to the patient population it is derived from is demonstrated in table 3: differences between intervals derived from hospital patients and those derived from general practitioners' patients occur, this is partly because of incomplete filtering of the pathological cases and also because of differences in the composition of the two populations. The differences between the serum albumen and protein intervals are the most marked.

### Conclusions

It is possible to derive reasonable reference intervals for a clinical chemical variable from a patient reference population provided that:

(1) The variable has an approximately Gaussian or gamma distribution.

(2) A choice can be made between these two models.

(3) The distribution is only partly distorted 'in the tails' by pathological observations.

(4) Many results are available (at least 1500).

(5) The precision of the determination is sufficiently large to allow sufficiently small classes in the histogram to 'resolve the model'.

(6) Results can be partitioned into groups which are expected to have similar 'reference limits' (sex, age etc.).

(7) It is recognized that the values established are only fit for a patient population that closely matches the reference population.

(8) A small computer is available, preferably one with plotting facilities.

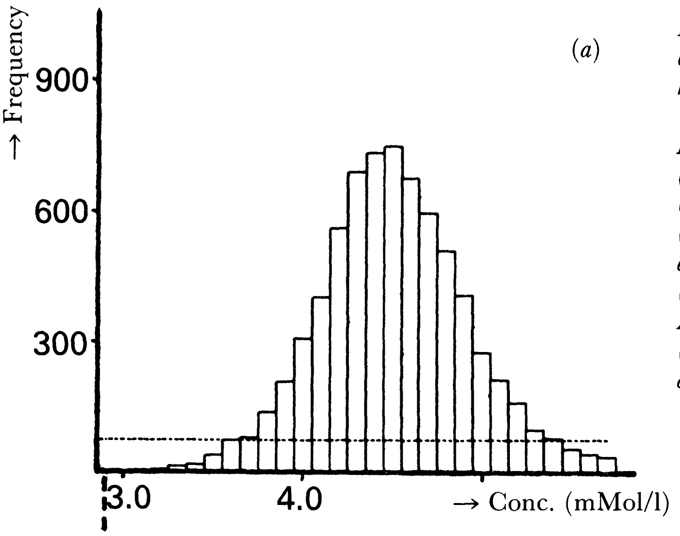

*Figure 5. A series of plots for potassium, for which a Gaussian distribution fits well. These plots are based on a mixture of hospital and general practitioner's patients.*

*None of these plots is smoothed.*
*(a) Histogram observed (with – – – – – threshold)*
*(b) Bhattacharya plot with regression line.*
*(c) Residuals from Bhattacharya plot, drawn only for the estimated reference interval.*
*(d) Plot to check the gamma model and regression line. (See Appendix B.)*
*(e) Residuals from plot (d) to facilitate the detection of systematic deviations from the gamma model.*

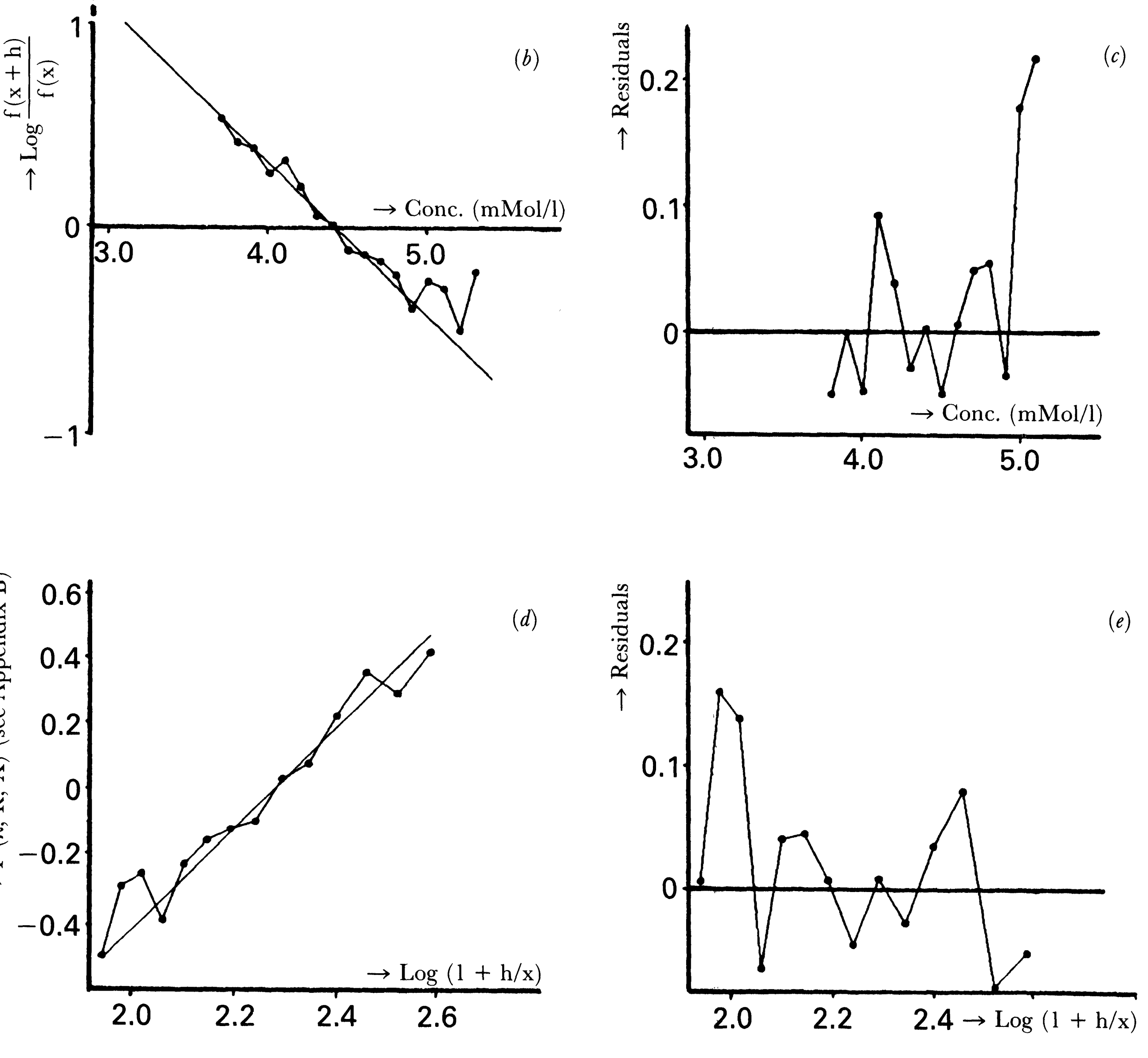

*Figure 6. Series of plots for ASAT, for which a gamma distribution fits well. These plots are based on a mixture of hospital and general practitioner's patients.*
*None of these plots is smoothed.*

*(a) Histogram observed (with - - - - - threshold).*
*(b) Bhattacharya plot with regression line.*
*(c) Residuals from Bhattacharya plot, drawn only for the estimated reference interval.*
*(d) Plot to check the gamma model and regression line. (See Appendix B.)*
*(e) Residuals from plot (d) to facilitate the detection of systematic deviations from the gamma model.*

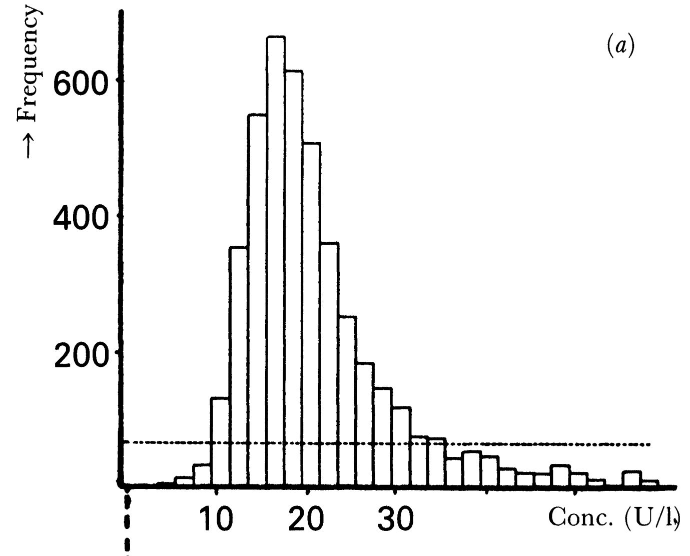

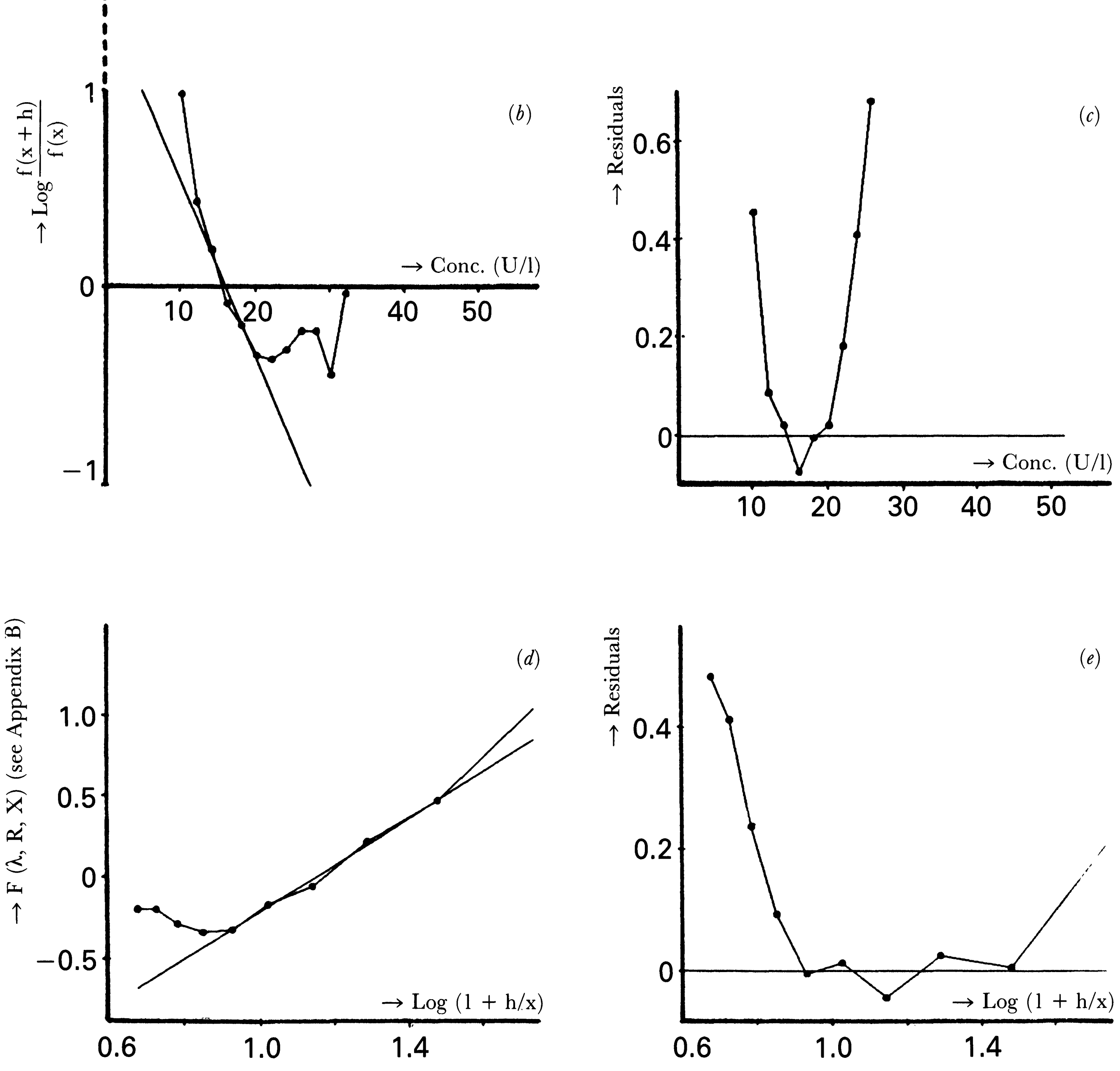

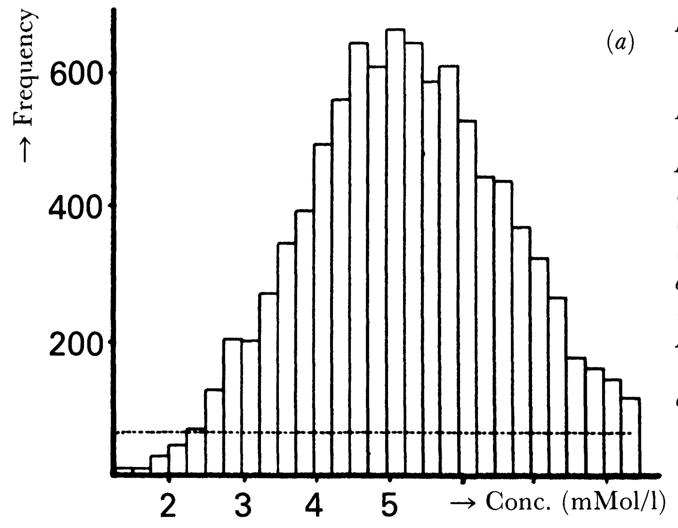

*Figure 7. Series of plots for cholesterol, for which a Gaussian distribution fits well. However, as the results should have been partitioned, the calculated reference interval (table 2) is nonsense.*

*These plots are based on a mixture of hospital and general practitioner's patients.*

*None of these plots is smoothed.*
*(a) Histogram observed (with - - - - - threshold).*
*(b) Bhattacharya plot with regression line.*
*(c) Residuals from Bhattacharya plot, drawn only for the estimated reference interval.*
*(d) Plot to check the gamma model and regression line. (See Appendix B.)*
*(e) Residuals from plot (d) to facilitate the detection of systematic deviations from the gamma model.*

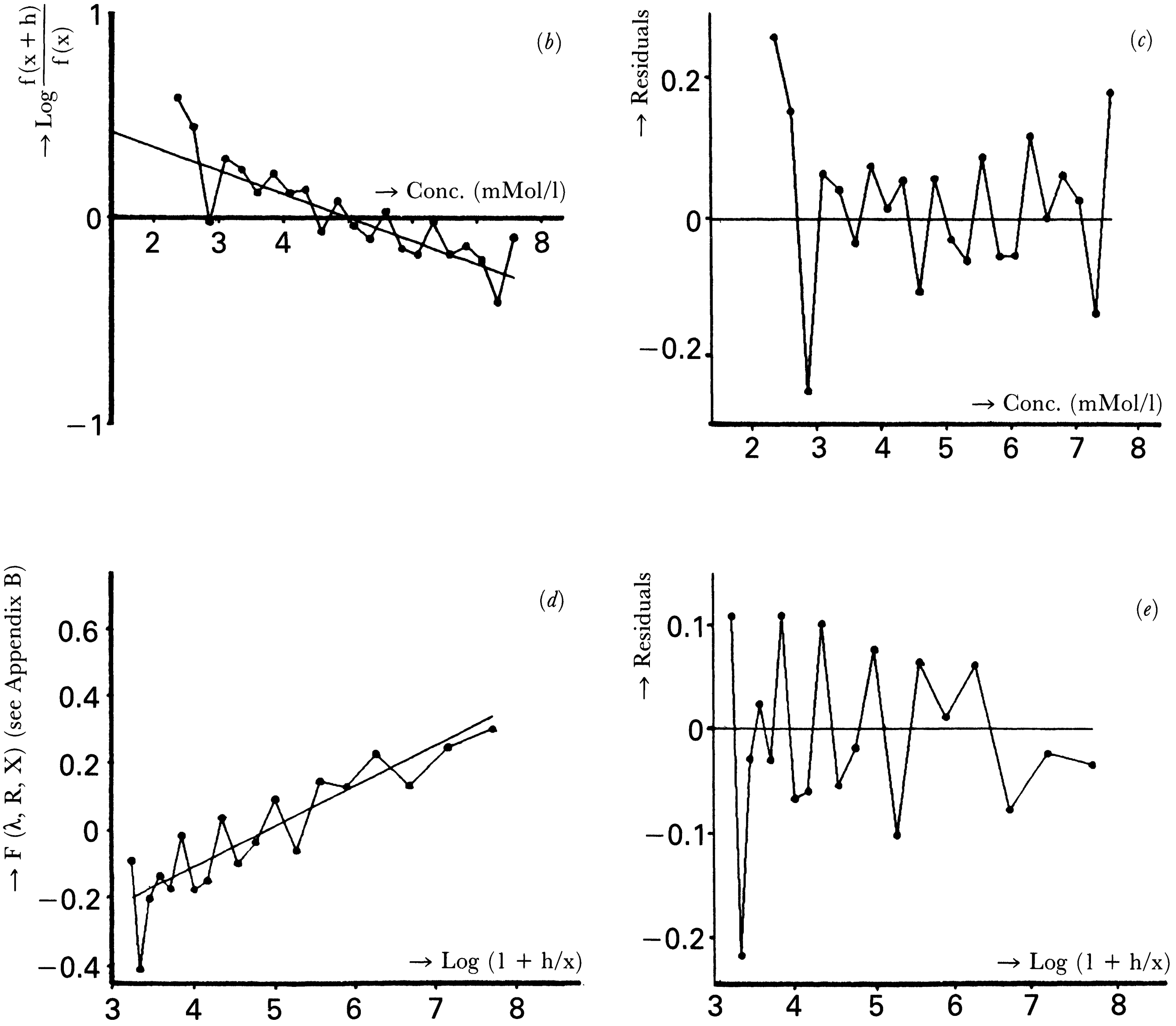

*Table 1. Results based on unpartitioned variables. These results are based on a mixture of hospital patients and general practitioner's patients. The variables were not partitioned according to sex, age, etc.[1]*

| Variable | N[2] | h[3] | Unity | Reference interval if Gaussian | R[4] | Reference interval if Gamma | One-sided reference interval | [5] |
|---|---|---|---|---|---|---|---|---|
| Sodium | 6695 | 1 | mmol/l | 134·6–143·6 | 3678 | 134·7–143·7 | | N |
| Potassium | 6778 | 0·1 | mmol/l | 3·66–5·09 | 146 | 3·71–5·14 | | N |
| Chloride | 3400 | 1 | mmol/l | 98·6–109·1 | 1457 | 98·6–109·3 | | N |
| Urea | 3246 | 0·5 | mmol/l | 1·92–7·50 | 12 | 2·59–8·21 | <7·64 | G |
| Creatinine | 3424 | 10 | μmol/l | 42–108 | 18 | 46–118 | <111 | G |
| LDH | 3433 | 10 | U/l | 127–277 | 30 | 140–287 | <274 | G? |
| ASAT (SGOT) | 4018 | 2 | U/l | 8·2–26·6 | 15 | 10·2–28·6 | <26·8 | G |
| ALAT (SGPT) | 3839 | 2 | U/l | 2·8–26·8 | 7 | 6·5–31·4 | <28·7 | G |
| Total bilirubin | 4085 | 1 | μmol/l | 1·7–11·6 | 8 | 3·1–13·1 | <12·0 | G |
| Calcium | 6610 | 0·04 | mmol/l | 2·178–2·624 | 440 | 2·186–2·635 | | N |
| Phosphate | 4347 | 0.1 | mmol/l | 0·67–1·49 | 27 | 0·73–1·57 | <1·49 | G |
| Total protein | 4087 | 2 | g/l | 59·3–77·2 | 218 | 59·7–77·9 | | N? |
| Albumen | 4141 | 2 | g/l | 35·3–50·6 | 110 | 35·6–51·7 | | N? |

*Table 2. Results based on variables that should be partitioned but were not.[1]*

| Variable | N[2] | h[3] | Unity | Reference interval if Gaussian | R[4] | Reference interval if Gamma | One-sided reference interval |
|---|---|---|---|---|---|---|---|
| AP* | 3464 | 10 | U/l | 28–130 | 11 | 41–142 | <132 |
| Cholesterol*† | 9499 | 0·25 | mmol/l | 2·31–8·13 | 13 | 2·92–9·00 | <8·39 |
| Iron†‡ | 4888 | 1·5 | μmol/l | 0–30·3 | 3 | 3·1–52·2 | <46·0 |

* Partitioning according to age is required.
† Partitioning according to sex is required.
‡ Partitioning according to sampling time is required.

*Table 3. Differences between reference intervals derived from hospital patients and those derived from general practitioner's patients.[1]*

| Variable | Reference interval for hospital patients | Width[7] | Reference interval[6] for GP patients | Width[7] | Unity | Difference[8] |
|---|---|---|---|---|---|---|
| Sodium | 134·9–144·8 | 9·9 | 134·8–142·7 | 7·9 | mmol/l | 22·5% |
| Potassium | 3·61–5·02 | 1·31 | 3·72–5·12 | 1·40 | mmol/l | 1·2% |
| Chloride | 98·1–109·0 | 10·9 | 99·0–109·3 | 10·3 | mmol/l | 6·2% |
| ASAT (SGOT) | 10·2–30·3 | 20·1 | 10·3–27·3 | 17·1 | U/l | 16·3% |
| ALAT (SGPT) | 5·6–31·9 | 26·3 | 7·6–29·7 | 22·1 | U/l | 16·6% |
| Total bilirubin | 2·9–13·9 | 11·0 | 3·3–12·5 | 9·1 | μmol/l | 18·4% |
| Calcium | 2·156–2·650 | 0·494 | 2·187–2·611 | 0·424 | mmol/l | 15·8% |
| Phosphate | 0·70–1·63 | 0·93 | 0·74–1·54 | 0·80 | mmol/l | 15·3% |
| Total protein | 56·8–78·3 | 21·5 | 60·3–76·8 | 16·4 | g/l | 28·5% |
| Albumen | 31·9–52·6 | 20·7 | 36·8–49·6 | 12·9 | g/l | 51·4% |

(1) The reference intervals are specified to one-tenth of the class width.
(2) Number of reference patients.
(3) h: Class width.
(4) R: R-parameter of gamma-distribution, number of degrees of freedom.
(5) In this column a suggestion is given for the choice of the distribution model (N = normal, G = gamma, ? = doubtful).
(6) The model suggested in table 1 is used here.
(7) Calculated from interval before rounding off.
(8) Calculated as a percentage of the width of the overall reference interval from table 1.

An alternative might be the method by Martin *et al.* [10 and 11], but this relies very heavily on 'magic computer calculations'; Martin *et al.*'s method has not yet been evaluated by the authors.

### Acknowledgements

The authors thank Hilko van der Voet for many critical discussions about this subject, Dr A. J. M. Naus for his clarifications, and Dr H. Verweij for critically reviewing the first manuscript.

### References

1. GRÄSBECK, R. and ALSTRÖM, T., *Reference Values in Laboratory Medicine* (Wiley, Chichester, 1981), 17.
2. IFCC, The theory of reference values. *Journal of Clinical Chemistry and Clinical Biochemistry*, **17** (1979), 337 (part 1), **22** (1984), 203 (part 2), **21** (1983), 749 (part 5), **20** (1982), 841 (part 6).
3. NAUS, A. J. M., *De berekening van referentiewaarden in de klinische chemie uit analyseresultaten van een patientenpopulatie.* (Thesis: Maastricht, The Netherlands, 1982).
4. HOFFMANN, R., *Clinical Chemistry*, **17** (1971), 456.
5. HOFFMANN, R., *Journal of the American Medical Association*, **185** (1963), 864.
6. NEUMANN, G., *Clinical Chemistry*, **14** (1968), 979.
7. PRYCE, J., *Lancet*, **2** (1960), 333.
8. BECKTEL, J., *Clinica Chimica Acta*, **28** (1970), 119.
9. BHATTACHARYA, C. G., *Biometrics*, **23** (1967), 115.
10. MARTIN, H. F., GUDZINOWICZ, B. J. and FANGER, H., *Normal Values in Clinical Chemistry* (Marcel Dekker, New York, 1975) 332.
11. MARTIN, H. F. *et al.*, Reference values based on populations accessible to hospitals, in GRÄSBECK, R. and ALSTRÖM, T., *Reference Values in Laboratory Medicine* (Wiley, Chichester, 1981), 233.
12. Wissenschaftliche Tabellen Geigy, *Teilband Statistik* (Ciba-Geigy AG, Basle, Switzerland, 1980), 204.
13. SAVITZKY, A. and GOLAY, J. E., *Analytical Chemistry*, **36** (1964), 1627.
14. STEINIER, J. *et al.*, *Analytical Chemistry*, **44** (1972), 2906.

### Appendix A

*Theoretical background to the Bhattacharya plot*

Although for a histogram the theory of the Bhattacharya plot is complicated (see Bhattacharya [9] or Naus [3]), a basic explanation of its background is:

The probability density of a normal distribution is defined by

$$f(x) = \frac{1}{\sigma\sqrt{2\pi}} e^{-\frac{1}{2}\left(\frac{x-\mu}{\sigma}\right)^2}$$

so

$$\frac{f(x+h)}{f(x)} = \frac{e^{-\frac{1}{2}\left(\frac{x+h-\mu}{\sigma}\right)^2}}{e^{-\frac{1}{2}\left(\frac{x-\mu}{\sigma}\right)^2}}$$

and

$$\log\frac{f(x+h)}{f(x)} = \frac{-[(x-\mu)^2 + 2h(x-\mu) + h^2 - (x-\mu)^2]}{2\sigma^2} = \frac{-h}{\sigma^2} + \frac{2h\mu - h^2}{2\sigma^2}$$

So the variance can be calculated from the slope of the regression line, and from the $x$-intercept ($= -y$-intercept/slope) we find $\mu$:

$$\sigma^2 = \frac{-h}{\text{slope}}$$

and

$$\mu = \frac{-y\text{-intercept}}{\text{slope}} + \frac{h}{2}.$$

Because we are using histograms a (Sheppard) discontinuity correction for $\sigma^2$ is necessary, so:

$$\sigma^2 = \frac{-h}{\text{slope}} - \frac{h^2}{12}$$

### Appendix B

*Estimating R and λ from a distribution sample given as a histogram*

A gamma distribution is given by

$$f(x) = \frac{\lambda^R}{\Gamma(R)} x^{R-1} e^{-\lambda x}$$

with

$$\Gamma(R) = \int_0^\infty x^{R-1} e^{-x}\,dx = (R-1)\int_0^\infty x^{R-2} e^{-x}\,dx = (R-1)\,\Gamma(R-1)$$

$(R > 0, \lambda > 0, x \geq 0)$

so

$$f(x+h) = \frac{\lambda^R}{\Gamma(R)} (x+h)^{R-1} e^{-\lambda(x+h)}$$

and

$$\log\frac{f(x+h)}{f(x)} = (R-1)\log(1 + h/x) - \lambda h \qquad (1)$$

where $h$ = class width.

This formula applies only if $h$ is infinitely small. If not, the following approximation could be used:

$$\log\frac{f(x+h)}{f(x)} = (R-1)\log(1 + h/x) - \lambda h + \frac{h^3}{12}\left[\frac{(R-1)\lambda}{x^2} - \frac{(R-1)(R-2)}{x^3}\right] \qquad (2)$$

This formula is derived as the first three terms of a Taylor series using some approximations. It is useless to calculate more terms from the series because of the other approximations that are necessary to get a formula that is relatively easy to work with.

(1) and (2) can be represented by:

$$(1) = Y \quad = AX + B \qquad (3)$$

$$(2) = Y \quad = A'X + B' + G(\lambda,R,X)$$

$$\text{or: } = Y - G(\lambda,R,X) = A'X + B' \qquad (4)$$

$$\text{with } Y = \log\frac{f(x+h)}{f(x)}$$

where $x = \log(1 + h/x)$, etc.

If using equation (3) a first estimation of $R$ and $\lambda$ is made from $A$ and $B$, and these estimates can be substituted in the left part of equation (4) to get better values from $A'$ and $B'$. By using equation (4) iteratively until convergence is reached, approximations of $R$ and $\mu$ are reached. These are too large and the estimates calculated from equation are too small. The average estimate

$$\bar{\lambda} = \frac{\lambda_{[3]} + \lambda_{[4]}}{2} \quad \text{and} \quad \bar{R} = \frac{R_{[3]} + R_{[4]}}{2}$$

are reasonably accurated (Naus [3]). If the sum of equations (3) and (4) are divided by 2:

$$\mathrm{Y} - \frac{G(\lambda,R,\mathrm{X})}{2} = \frac{A + A'}{2}\mathrm{X} + \frac{B + B'}{2}$$

or

$$F(\lambda,R,\mathrm{X}) = \frac{A + A'}{2}\mathrm{X} + \frac{B + B'}{2}.$$

So a plot of $F(\lambda,R,X)$ (with $\lambda$ and $R$: the least estimates for the parameters, based on (4)) against $X$, that is,

$$\log\frac{f(x+h)}{f(x)} - \frac{h^3}{24}\left[\frac{(R-1)\lambda}{x^2} - \frac{(R-1)(R-2)}{x^3}\right]$$

against $\log(1 + h/x)$

will give a straight line for a gamma distribution, with $R$ and $\lambda$ as estimates. Using the plot, $R$ can be calculated from the slope and lambda from the intercept of the regression line. A plot of residuals can be drawn to detect systematic deviations from the gamma model. Since the regression is using weightings, the interpretation of the results must also be weighted. To facilitate this, both plots are made for the calculated reference interval only, where no extreme low frequencies are present.

### Appendix C

*Finding gamma fractiles from a chi-square distribution*

Chi-square distributions have the same relationship to gamma distributions as standard normal distributions have to normal ones. So from every gamma distribution a chi-square version can be calculated.

Areas under the gamma distribution curve are given by

$$\text{gamma}(\mathrm{X},\lambda,R) = \frac{1}{\Gamma(R)}\int_0^x \lambda^R t^{R-1} e^{-\lambda t} dt.$$

By substitution of $t = p/(2\lambda)$ we get:

$$\frac{1}{\Gamma(R)}\int_0^{2\lambda x} (\tfrac{1}{2})^R p^{R-1} e^{-\frac{1}{2}p} dp.$$

And this is exactly the area under the curve of a chi-square distribution with 2R degrees of freedom, from 0 to $2\lambda x$.

So gamma $(x;\lambda,R) = \chi^2(2\lambda x;2R)$.

By similar reasoning we find $\chi^2(x;\nu)$ = gamma $(x;1/2, \nu/2)$:

$$\chi^2(x;\nu) = \frac{1}{\Gamma(\nu/2)}\int_0^x (\tfrac{1}{2})^{\frac{\nu}{2}} t^{\frac{\nu}{2}-1} e^{-\frac{t}{2}} dt$$

which is the formula for the area under the gamma-distribution curve with $R = \nu/2$ and $\lambda = 1/2$.

If $R > 4$, an acceptable approximation of the 0.025 and 0.975 fractiles is given by

$$\frac{(\sqrt{4R} \pm 1.96)^2}{4\lambda}$$

which formula is based on a usual approximation of chi-square fractiles [12].

